# Diagnostic accuracy of pre-operative breast magnetic resonance imaging (MRI) in predicting axillary lymph node metastasis: variations in intrinsic subtypes, and strategy to improve negative predictive value—an analysis of 2473 invasive breast cancer patients

**DOI:** 10.1007/s12282-023-01488-9

**Published:** 2023-07-27

**Authors:** Shu-Tian Chen, Hung-Wen Lai, Julia Huei-Mei Chang, Chiung-Ying Liao, Tzu-Cheng Wen, Wen-Pei Wu, Hwa-Koon Wu, Ying-Jen Lin, Yu-Jun Chang, Shou-Tung Chen, Dar-Ren Chen, Hsin-I Huang, Che-Lun Hung

**Affiliations:** 1 Department of Diagnostic Radiology, Chang Gung Memorial Hospital - Chiayi Branch, Chiayi, Taiwan; 2 Institute of Biomedical Informatics, National Yang Ming Chiao Tung University, No.155, Sec. 2, Linong St., Beitou Dist., Taipei, 11221 Taiwan; 3 Department of Biomedical Imaging and Radiological Sciences, National Yang Ming Chiao Tung University, Taipei, Taiwan; 4 School of Medicine, National Yang Ming Chiao Tung University, Taipei, Taiwan; 5 Endoscopy and Oncoplastic Breast Surgery Center, Changhua Christian Hospital, 135 Nanxiao Street, Changhua, 500 Taiwan; 6 Division of General Surgery, Changhua Christian Hospital, Changhua, Taiwan; 7 Comprehensive Breast Cancer Center, Changhua Christian Hospital, Changhua, Taiwan; 8 Tumor Center, Changhua Christian Hospital, Changhua, Taiwan; 9 Department of Radiology, Changhua Christian Hospital, Changhua, Taiwan; 10 Big Data Center, Changhua Christian Hospital, Changhua, Taiwan; 11 Kaohsiung Medical University, Kaohsiung, Taiwan; 12 Division of Breast Surgery, Yuanlin Christian Hospital, Yuanlin, Taiwan; 13 School of Medicine, Chung Shan Medical University, Taichung, Taiwan; 14 Department of Pathology, Changhua Christian Hospital, Changhua, Taiwan; 15 Department of Information Management, National Sun Yat-Sen University, Kaohsiung, Taiwan; 16 We-Sing Breast Hospital, Kaohsiung, Taiwan; 17 Department of Computer Science and Communication Engineering, Providence University, Taichung, Taiwan

**Keywords:** Breast magnetic resonance imaging (MRI), Lymph node metastasis, Intrinsic subtype, Sentinel lymph node biopsy (SLNB), Negative predictive value (NPV)

## Abstract

**Background:**

The value and utility of axillary lymph node (ALN) evaluation with MRI in breast cancer were not clear for various intrinsic subtypes. The aim of the current study is to test the potential of combining breast MRI and clinicopathologic factors to identify low-risk groups of ALN metastasis and improve diagnostic performance.

**Material and methods:**

Patients with primary operable invasive breast cancer with pre-operative breast MRI and post-operative pathologic reports were retrospectively collected from January 2009 to December 2021 in a single institute. The concordance of MRI and pathology of ALN status were determined, and also analyzed in different intrinsic subtypes. A stepwise strategy was designed to improve MRI-negative predictive value (NPV) on ALN metastasis.

**Results:**

2473 patients were enrolled. The diagnostic performance of MRI in detecting metastatic ALN was significantly different between intrinsic subtypes (*p* = 0.007). Multivariate analysis identified tumor size and histologic type as independent predictive factors of ALN metastases. Patients with HER-2 (MRI tumor size ≤ 2 cm), or TNBC (MRI tumor size ≤ 2 cm) were found to have MRI–ALN-NPV higher than 90%, and these false cases were limited to low axillary tumor burden.

**Conclusion:**

The diagnostic performance of MRI to predict ALN metastasis varied according to the intrinsic subtype. Combined pre-operative clinicopathologic factors and intrinsic subtypes may increase ALN MRI NPV, and further identify some groups of patients with low risks of ALN metastasis, high NPV, and low burdens of axillary disease even in false-negative cases.

## Introduction

Axillary lymph node (ALN) staging remains critical in the management of patients with breast cancer; it helps determine the clinical stage, treatment plan, and prognosis [1]. The standard of reference and the most accurate way for evaluating ALN status in breast cancer patients is surgical lymph node biopsy, either axillary lymph node dissection (ALND) or sentinel lymph node biopsy (SLNB) [2, 3]. SLNB, which has been associated with less morbidity, has gradually become the dominant surgical ALN evaluation procedure in patients with clinical node-negative primary operable breast cancer [4–9].

However, SLNB is not a risk-free procedure, which is related to about 6% axillary lymph edema rate and up to 10% morbidity risk [10, 11]. Non-invasive lymph node evaluation methods that result in lower morbidity than surgical ALN biopsy without compromising disease control remain the goal of modern imaging studies. The non-invasive imaging modalities for assessing ALNs are rapidly evolving, and magnetic resonance imaging (MRI) has been one of the potentially promising tools. A meta-analysis revealed the pooled diagnostic sensitivity and specificity of MRI to detect ALN metastasis in patients with breast cancer were 0.77 (95% confidence interval [CI] 0.75**–**0.80) and 0.90 (95% CI 0.89**–**0.91), respectively [12]. Recent studies showed the negative predictive value (NPV) was around 80% and accuracy was 70–80% for MRI to detect ALN metastasis in breast cancer patients [13–26], which is acceptable but remained space to improve.

In the past 10 years, breast cancer diagnosis and treatment have been greatly influenced by the concept of different intrinsic subtypes [27], which have different patterns of disease presentation, metastatic spread, and response to treatment [28, 29]. However, there is limited information about the diagnostic accuracy of breast MRI in the prediction of ALN metastasis regarding different intrinsic subtypes. We hypothesized that the intrinsic subtype might influence the diagnostic accuracy of MRI in the prediction of ALN metastasis, and by combining pre-operative clinicopathologic factors, intrinsic subtypes, and MRI findings, we could further identify some relatively low-risk groups of patients with invasive breast cancer and improving the diagnostic performance of MRI for ALN.

## Material and methods

### Patients

Women with primary operable invasive breast cancer who underwent breast surgery during the period of January 2009 to December 2021 were retrospectively recruited in this cohort study. Patients were systemically excluded from the study if there was no pre-operative MRI, non-invasive breast cancer, received a neoadjuvant treatment (chemotherapy, hormone therapy or radiotherapy), or had a locoregional recurrence, or if the axillary status was not mentioned in the MRI report or pathology report. The clinicopathologic factors gathered from the database include age, tumor location, biopsy method, pathologic tumor size, histology, tumor grade, status of estrogen receptor (ER), progesterone receptor (PR), human epithelial growth factor receptor 2 (HER-2) expression, and Ki-67 percentage. The study was approved by our Institutional Review Board and granted a waiver of informed consent.

### Diagnostic accuracy of breast MRI to predict ALN metastasis in different intrinsic subtypes

Diagnostic performance parameters (sensitivity, specificity, positive predictive value (PPV), NPV, and accuracy) were calculated for breast MRI. In the subgroup analysis, these diagnostic performance parameters were calculated for each intrinsic subtype using immunohistochemistry (IHC) surrogate markers [30]. The subtypes were Luminal A (ER > 1% positive, PR ≥ 20% positive, HER-2 negative, Ki-67% ≤ 14%), Luminal B1 (ER > 1% positive, and/or PR < 20% positive, HER-2 negative, Ki-67% > 14%), Luminal B2 (ER > 1% positive, and/or PR > 1% positive, HER-2 positive, Ki-67% ≤ 14%), HER-2 (ER and PR negative, HER-2 positive), and triple-negative breast cancer (TNBC) (ER/PR/HER-2 negative).

### MRI protocols

MR imaging was performed with a 3.0 Tesla MRI machine (Siemens MAGNETOM Verio, Munich, Germany). All patients were imaged in the prone position with both breasts placed into a dedicated 16-channel breast coil. MR imaging protocols included the following: bilateral axial turbo-spin-echo fat-suppressed T2-weighted imaging (TR/TE 4630/70 ms; field of view 320 mm; slice thickness 3 mm; number of excitations (1), axial turbo-spin-echo T1-weighted imaging (TR/TE 736/9.1 ms; field of view 320 mm; slice thickness 3 mm; number of excitations (1). Dynamic contrast-enhanced MR images (DCE-MRI) were obtained with a three-dimensional fat-suppressed volumetric interpolated breath-hold examination (VIBE) sequence with parallel acquisition once before and five times after a bolus injection of gadobenate dimeglumine (0.1 mmol/kg). Both breasts were examined in the transverse plane at 60 s intervals in each phase of the dynamic studies. The dynamic MRI parameters were as follows: TR/TE 4.36/1.58 ms; field of view 320 mm; slice thickness 1 mm.

### Evaluation of axillary lymph nodes via MRI

Three radiologists with 37, 18, and 12 years of breast imaging experience performed the breast MRI interpretation and made the reports. The ALNs were assessed by nodal morphology and size on T2-weighted and contrast-enhanced T1-weighted sequences. Morphology criteria of pathologic nodes were considered when there were one or more of the following features: cortical thickening greater than 3 mm, abnormal lymph node shape (round or not uniform), completely/partially effaced fatty hilum, or asymmetry compared with the contralateral side [31].

### Statistical analyses

Data were expressed as mean ± standard deviation for continuous variables and numbers (percentage) for categorical or ordinal variables. Differences in the MRI ALN diagnostic performance regarding true-positive, false-positive, true-negative, and false-negative numbers among intrinsic subtypes were evaluated by Kruskal–Wallis test for non-normal distribution. Significant predictors in the univariate analysis were included in a multivariate logistic regression model to identify the most important predictors. The incidence of metastatic ALN, the NPV of MRI, the nodal status distribution, and the average number of false-negative ALNs were calculated to assess the MRI efficacy after subgrouping by tumor size on pathology and imaging, as well as by intrinsic subtype. These covariates were chosen based on multivariate findings. Statistical analyses were performed by statistical experts using Statistical Product and Service Solutions (SPSS) for Windows (Version 19.0, SPSS Inc, Chicago, IL).

## Results

### Study participants

**Fig. 1:**
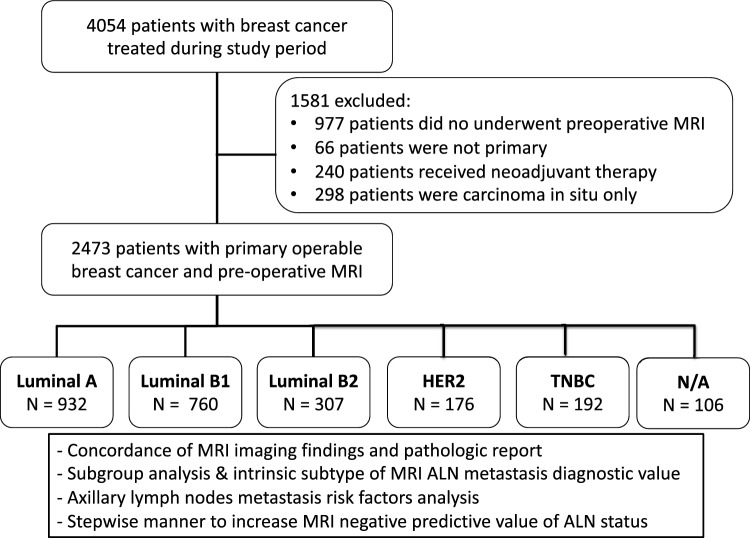
Study design and patients’ allocation of current MRI axillary lymph node diagnostic performance evaluation. ALN: axillary lymph node. HER-2: human epidermal growth factor receptor 2 TNBC: triple-negative breast cancer

A total of 2473 patients with primary operable invasive breast cancer who underwent pre-operative breast MRI evaluation and post-operative pathologic ALN biopsy results were enrolled in the current study (Fig. 1). Among them, 861 (34.8%) patients had pathologically confirmed metastatic ALNs, and 1612 (65.2%) had negative ALNs. According to intrinsic subtype classifications, there were 932 (39.4%) Luminal A, 760 (32.1%) Luminal B1, 307 (13%) Luminal B2, 176 (7.4%) HER-2, and 192 (8.1%) TNBC patients. The demographic data and histological results of the 2473 patients are summarized in Table 1.

**Table 1:** Demographic data and tumor characteristics of the 2473 Patients

N = 2473	N (%), MEANS ± SD
Age, Y	54.0 ± 11.2
Location	
Right	1194 (48.3)
Left	1279 (51.7)
Biopsy method (N/A = 37)	
Us-guided core needle biopsy	2302 (94.5)
Stereotactic vacuum-assisted biopsy	53 (2.2)
Excisional biopsy	72 (3.0)
Fine needle aspiration	5 (0.2)
Stereotactic core needle biopsy	4 (0.2)
Tumor size, cm	2.3 (1.6)
Lymph node	
Positive	861 (34.8)
Negative	1612 (65.2)
Lymph node stage	
N0	1612 (65.2)
N1	644 (26.0)
N2	142 (5.7)
N3	75 (3.0)
Stage	
I	1013 (41.0)
II	1192 (48.2)
III	253 (10.2)
IV	15 (0.6)
Pathology (N/A = 17)	
IDC	2214 (90.1)
ILC	123 (5.0)
Other	119 (4.8)
Grade (N/A = 54)	
I	528 (21.8)
II	1345 (55.6)
III	546 (22.6)
ER (N/A = 16)	
Positive	2035 (82.8)
Negative	422 (17.2)
PR (N/A = 17)	
Positive	1815 (73.9)
Negative	641 (26.1)
HER-2 (N/A = 82)	
Positive	468 (19.6)
Negative	1923 (80.4)
Subtype (N/A = 106)	
Luminal A	932 (39.4)
Luminal B1	760 (32.1)
Luminal B2	307 (13.0)
HER-2( +)	176 (7.4)
TNBC	192 (8.1)
Ki-67 (N/A = 216)	
≦14	942 41.7)
> 14	1315 (58.3)

*N/A* not available, *ER* estrogen receptor, *PR* progesterone receptor, *HER-2* human epidermal growth factor receptor 2, *TNBC* triple-negative breast cancer, *IDC* invasive ductal carcinoma, *ILC* invasive lobular carcinoma

### MRI ALN prediction in different intrinsic subtypes

The diagnostic performance of breast MRI to predict ALN metastasis was calculated: the sensitivity was 63.2% (544/861), specificity 68.5% (1104/1612), NPV 77.7% (1104/1421), PPV 51.7% (544/1052), and the overall accuracy was 66.6% (1648/2473) (Table 2).

**Table 2:** Diagnostic performance of MRI on axillary lymph node metastasis per molecular subtype and subgroup analysis

N = 2473	Total	Luminal A	Luminal B1	Luminal B2	HER-2	TNBC
Sensitivity %(95% CI)	63.2 (60–67)	53.8 (47–60)	65.5 (61–72)	67.9 (60–77)	62.5 (49–75)	77.8 (64–88)
Specificity % (95% CI)	68.5 (67–71)	76.2 (73–80)	67.1 (63–73)	63.0 (55–70)	57.1 (48–66)	57.6 (49–66)
PPV % (95% CI)	51.7 (48–54)	48.5 (42–54)	58.2 (54–65)	58.7 (50–66)	40.7 (30–52)	42.9 (33–53)
NPV % (95% CI)	77.7 (77–81)	79.7 (77–83)	73.5 (70–78)	71.7 (65–80)	76.4 (66–85)	86.4 (77–93)
Accuracy % (95% CI)	66.6 (65–69)	69.5 (67–73)	66.4 (64–71)	65.1 (60–71)	58.9 (51–66)	63.4 (56–70)

	N0	N1	N2	N3	N0 + N1	N2 + N3
Sensitivity % (95% CI)		57.1 (54–62)	78.2 (70–85)	86.7 (76–93)	57.1 (54–62)	81.1 (75–86)
Specificity % (95% CI)	68.5 (67–71)				68.5 (67–71)	
PPV % (95% CI)		100 (99–100)	100 (97–100)	100 (94–100)	42.1 (38–45)	100 (98–100)
NPV % (95% CI)	100 (100–100)				80.0 (79–83)	

*TNBC* triple-negative breast cancer, *HER-2* human epidermal growth factor receptor 2, *CI* confidence interval, *NPV* negative predictive value, *PPV* positive predictive value

The accuracy of breast MRI for detecting metastatic ALNs in different intrinsic subtypes was evaluated and is summarized in Table 2. The NPV was highest in TNBC (86.4%), and lowest in Luminal B2 (71.7%). The sensitivity was highest in the TNBC group (77.8%), Luminal A was associated with the highest specificity (76.2%), and the highest PPV was in the Luminal B2 group (58.7%). The distribution of true-positive, true-negative, false-positive, and false-negative between intrinsic groups was significantly different (*p* = 0.007).

### Axillary lymph node tumor burden and MRI predictive value

The sensitivity of MRI ALN metastasis prediction increased significantly from 57.1% in N1 (1–3 positive nodes) to 78.2% in N2 (4–9 positive nodes), and 86.7% N3(≥ 10 positive nodes). In patients with no to low axillary tumor burden (N0 + N1), the sensitivity of MRI to detect positive ALN was 57.1%, and in patients with high axillary tumor burden (N2 + N3), the sensitivity increased to 81.1% (Table 2).

### Prediction of ALN metastasis by clinicopathologic biomarkers

Using univariate analysis, we found that pathologic tumor size, MRI tumor size, histologic type, histologic grade, ER, PR, and Ki-67 were statistically significant predictors of ALN metastasis. Multivariate analysis identified pathologic tumor size (Odds ratio (OR) = 1.48), MRI tumor size (OR = 1.19), histologic type (non-invasive ductal carcinoma (IDC) versus IDC, OR = 0.47), higher histologic grade (OR = 1.33), and PR (OR = 1.91) were independent predictive factors of ALN metastasis (Table 3).

**Table 3:** Risk factors for axillary lymph node metastasis in breast cancer patients

	Univariate analysis			Multivariate analysis		
	Odds ratio	95% CI	*P*-value	Odds ratio	95% CI	*P*-value
Age	1.00	0.99–1.00	0.72			
Pathologic tumor size (invasive, cm)	1.58	1.48–1.69	< 0.01	1.48	1.34–1.63	< 0.01
MRI tumor size (invasive, cm)	1.29	1.23–1.35	< 0.01	1.19	1.11–1.28	< 0.01
Histology type (Non-IDC) vs. IDC	0.59	0.43–0.80	< 0.01	0.47	0.32–0.69	< 0.01
Histological Grade (II, III) vs. I	1.79	1.43–2.25	< 0.01	1.33	1.02–1.73	0.04
ER (positive) vs. negative	1.31	1.04–1.64	0.02	1.52	0.90–2.59	0.12
PR (positive) vs. negative	1.26	1.04–1.52	0.02	1.91	1.31–2.80	< 0.01
HER-2 (positive) vs. negative	1.20	0.97–1.48	0.09			
Ki -67% (> 14) vs. ≦14	1.48	1.24–1.76	< 0.01	1.16	0.93–1.46	0.20

*IDC* invasive ductal carcinoma, *ER* estrogen receptor, *PR* progesterone receptor, *HER-2* human epidermal growth factor receptor2, *CI* Confidence interval

*p < 0.05

### Prevalence of ALN metastasis and NPV of MRI by combining clinicopathologic and imaging factors

The ALN-positive rate was 29.5% (275/932) in Luminal A, 41.2% (313/760) in Luminal B1, 43.6% (134/307) in Luminal B2, 30.1% (53/176) in HER-2, and 29.7% (57/192) in TNBC. Based on the results of multivariate analysis (pathologic & MRI tumor size), four individual risk groups of nodal involvement for each intrinsic subtype were generated (Table 4). The prevalence of ALN metastases, NPV, average false-negative (FN) LN, and distributions of lymph nodes (N1, N2, and N3) were further summarized and correlated to different intrinsic subtypes and tumor size for further evaluation. Patients with HER-2 (MRI tumor ≤ 2 cm), or TNBC (MRI tumor size ≤ 2 cm, MRI tumor size ≤ 3 cm, and pathologic tumor size ≤ 3 cm) were found to have MRI-ALN-NPV higher than 90%, and these FN cases were limited to low axillary tumor burden (N1, Table 4).

**Table 4:** Correlation of intrinsic subtype, tumor size, lymph node metastasis, and MRI-negative predictive value

	Prevalence of LN metastasis N (%)	NPV of MRI %(95%CI)	Average FN LN^a^	N1 N (%)	N2 N (%)	N3 N (%)
Total	861/2473 (34.8)	77.7 (76.7–81.1)	2.3	276 (87.1)	31 (9.8)	10 (3.2)
Luminal A	275/932 (29.5)	79.7 (76.7–83.3)	1.7	116 (91.3)	11 (8.7)	0
P size ≤ 2 cm	107/557 (19.2)	86.6 (82.7–89.9)	1.5	58 (95.1)	3 (4.9)	0
P size ≤ 3 cm	203/792 (25.6)	82.5 (78.9–85.8)	1.5	96 (94.1)	6 (5.9)	0
MRI size ≤ 2 cm	53/314 (16.9)	76.4 (67.9–83.6)	1.5	30 (96.8)	1 (3.2)	0
MRI size ≤ 3 cm	123/580 (21.2)	85.6 (81.7–88.9)	1.5	59 (95.2)	3 (4.8)	0
Luminal B1	313/760 (41.2)	73.5 (70.0–78.0)	2.7	91 (84.3)	10 (9.3)	7 (6.5)
P size ≤ 2 cm	95/339 (28.0)	81.7 (75.6–86.8)	1.6	40 (95.2)	2 (4.8)	0
P size ≤ 3 cm	195/570 (34.2)	78.4 (73.3–82.9)	1.5	72 (94.7)	4 (5.3)	0
MRI size ≤ 2 cm	35/173 (20.2)	88.1 (80.9–93.4)	1.7	18 (94.7)	1 (5.3)	0
MRI size ≤ 3 cm	131/424 (30.9)	79.6 (74.0–84.5)	1.7	54 (94.7)	2 (3.5)	1 (1.8)
Luminal B2	134/307 (43.6)	71.7 (64.7–79.8)	2.1	38 (88.4)	4 (9.3)	1 (2.3)
P size ≤ 2 cm	35/138 (25.4)	83.5 (73.5–90.9)	2.5	14 (87.5)	1 (6.3)	1 (6.3)
P size ≤ 3 cm	90/240 (37.5)	76.9 (68.3–84.0)	2.0	31 (91.2)	2 (5.9)	1 (2.9)
MRI size ≤ 2 cm	8/42 (19.0)	84.4 (67.2–94.7)	2.2	4 (80.0)	1 (20.0)	0
MRI size ≤ 3 cm	49/145 (33.8)	79.8 (69.6–87.7)	2.6	18 (85.7)	2 (9.5)	1 (4.8)
HER-2	53/176 (30.1)	76.4 (66.2–84.8)	2.4	15 (83.3)	2 (11.1)	1 (5.6)
P size ≤ 2 cm	14/87 (16.1)	82.8 (69.1–90.9)	1.3	8 (100.0)	0	0
P size ≤ 3 cm	32/136 (23.5)	79.7 (69.7–87.3)	1.6	13 (92.9)	1 (7.1)	0
MRI size ≤ 2 cm	2/18 (11.1)	90.1 (74.6–98.1)*	1.0	1 (100.0)	0	0
MRI size ≤ 3 cm	11/56 (19.6)	78.9 (62.7–90.4)	1.3	7 (100.0)	0	0
TNBC	57/192 (29.7)	86.4 (77.4–92.8)	3.7	12 (80.0)	2 (13.3)	1 (6.7)
P size ≤ 2 cm	14/90 (15.6)	92.5 (81.8–97.9)*	1.6	6 (100.0)	0	0
P size ≤ 3 cm	33/147 (22.4)	91.7 (82.7–96.9)*	1.3	8 (100.0)	0	0
MRI size ≤ 2 cm	5/35 (14.3)	92.3 (74.9–99.1)*	1.0	2 (100.0)	0	0
MRI size ≤ 3 cm	16/81 (19.8)	92.3 (81.5–97.9)*	1.4	5 (100.0)	0	0

*NPV* negative predict value, *FN* false negative, *LN* lymph node, *P size* pathological size, *TNBC* triple-negative breast cancer

*NPV > 90%

## Discussion

Our current study enrolled 2473 primary operable invasive breast cancer patients with detailed pre-operative breast MRI evaluation and post-operative ALN pathologic results for diagnostic accuracy analysis. We analyzed the performance of MRI in ALN metastatic status evaluation and found a significant difference between intrinsic subtypes. Factors related to ALN metastasis were also analyzed with univariate and multivariate analyses. We found that in patients with pre-operative MRI showing negative ALN metastasis and small tumor size, some intrinsic subtypes (HER-2 and TNBC) patients were associated with high NPV, relatively low risk of FN ALN, and even in FN cases were limited to low axillary tumor burden (N1).

Owing to more effective pre-operative evaluation and locoregional adjuvant therapies, the potential risks of axillary surgery may outweigh its actual benefits, especially in early-stage breast cancer patients treated with breast-conserving surgery [6, 32]. In 1994, Giuliano reported that SLNB is a highly reliable modality in axillary staging [3]. Since then, there has been a trend towards minimizing invasive staging and treatment of the axilla in clinically node-negative breast cancer patients owing to increased arm morbidity and decreasing quality of life after ALND [8]. In our current cohort, the positive ALN rate was only about a third (34.8% (861/2473), Table 1) in patients with primary operable invasive breast cancer patients, which echoed the need for less-invasive ALN evaluation and surgical treatment policy in breast cancer of screening era.

In the past decade, many studies were designed to investigate non-invasive imaging staging of the axilla [33, 34] to decrease surgical morbidity and, thus, improve patients’ quality of life. MRI is widely used on breast cancer patients for pre-operative assessment of disease extent and ALN status, screening of the contralateral breast, and evaluation of post-neoadjuvant treatment outcome [35, 36]. In the current study, we found that breast MRI is associated with 63.2% sensitivity, 68.5% specificity, 77.7% NPV, 51.7% PPV, and 66.6% accuracy. These results were consistent with previous literature reported series summarized in Table 5, and overall, MRI is associated with an NPV of around 68.4% ~ 85%, and accuracy in a range of 66.6% ~ 90%. When patients were segregated into different axillary disease extent, the sensitivity increased from 57.1% (N1) in low tumor burden cases to 78.2% (N2) or 86.7% (N3) in high tumor burn patients (Table 2). It meant that in patients with no to low axillary tumor burden (N0 + N1), the sensitivity of MRI to detect positive ALN metastasis was 57.1% (PPV 42.1%), and in patients with high axillary tumor burden (N2 + N3), the sensitivity significantly increased to 81.1% with a 100% PPV100%.

**Table 5:** MRI diagnostic performance on axillary lymph node in current study combined with literature review

Author	Journal/Year	Patient numbers	Reference standard	Sensitivity (%)	Specificity (%)	NPV (%)	PPV (%)	Accuracy (%)
Yoshimura et al. [13]	Breast Cancer/1999	202	ALND	79	93	87	89	88
Kvistad et al. [14]	Eur Radiol/2000	65	ALND	83	90	90	83	88
Orguc et al. [15]	Balkan Med J/2012	155	ALND	89	14	80*	21.4*	Not reported
He et al. [16]	Eur J Radiol/2012	136	ALND	33.3–86.5	95.2–98.2	1.9–16.7	66.7–82.6	18.5–96.2
Scaranelo et al. [17]	Radiology/2012	61	ALND/SLNB	88.4	82.4	94.7	69.4	85
Hwang et al. [18]	J Breast Cancer/2013	349	ALND/SLNB	47.8	88.7	82.6	60.2	77.9
Hieken et al. [19]	Surgery/2013	505	ALND/SLNB	54.2	78.2	75.7	57.7	69.7
Abe et al. [20]	Acad Radiol/2013	50	ALND/SLNB	60	79	81	59	74
An et al. [21]	Nuklearmedizin/2014	132	ALND	67.5	78	79.2	65.9	74
Arslan et al. [22]	Springerplus/2016	35	SLNB	73.3	95	82.6	91.7	85.7
Hyun et al. [23]	Eur J Radiol/2016	425	ALND/SLNB	51.3	92.2	83.3	71.4	80.9
Barco et al. [24]	Clin Transl Oncol/2016	1351	ALND/SLNB	29.8	96.6	68.4	84.9	Not reported
Atallah et al. [25]	Breast J/2020	169	ALND/SLNB	70.3	87.5	76.2	83.3	Not reported
Zhao et al. [26]	Eur J Radiol/2020	265	ALND/SLNB	72.7	87.1	71	94.3	Not reported
Chen et al.	Current study	2473^#^	ALND/SLNB	63.2 (544/861)	68.5 (1104/1612)	77.7 (1104/1421)	51.7 (544/1052)	66.6 (1648/2473)

*MRI* magnetic resonance imaging, *PPV* positive predictive value, *NPV* negative predictive value, *ALND* axillary lymph node dissection, *SLNB* sentinel lymph node biopsy

*Calculated parameters

^#^All these 2473 cases were primary operable invasive breast cancer patients

The incorporation of breast cancer “intrinsic subtype” information into clinical breast cancer assessment and treatment planning became an important step toward personalized medical care [30]. Hence, examining MRI results among different breast cancer intrinsic subtypes is an emerging area of research. Our study demonstrated that the diagnostic performance of ALN evaluation via MRI was significantly different among intrinsic subtypes (*p* = 0.007, Table 2). The luminal A (69.5%) breast cancer was associated with the highest overall accuracy while HER-2 (58.9%) was the lowest. The overall NPV of MRI was 77.7%, and this NPV could be increased to “86.4% and 79.7%” in “TNBC and Luminal A,” and dropped to “71.7% and 73.5%” in Luminal B2 & Luminal B1 type breast cancer. These variations in the diagnostic performance of MRI in ALN evaluation, which were important and rarely reported before, reminded clinical physicians the awareness of the impact of intrinsic subtypes on the accuracy of imaging interpretations.

To improve the diagnostic accuracy of pre-operative non-invasive imaging, we tried to identify clinicopathologic factors related to ALN metastasis with univariate and multivariate analysis. Pathologic tumor size (odds ratio, *OR* = 1.48), MRI tumor size (*OR* = 1.19), higher histologic grade (grade II, III versus I, *OR* = 1.33), and PR positivity (*OR* = 1.91) were significant independent risk factors (Table 3). These results were consistent with previous studies [37–39] showing that tumor size, either from pathology reports or from MRI reports, was an important independent predictive factor of ALN metastasis. By combining IHC biomarkers (intrinsic subtypes) and anatomical features (tumor size) that associated with ALN involvement, we further improved the NPV of ALN by MRI up to more than 90% in some groups of patients. Patients with HER-2 with pre-operative MRI tumor (≤ 2 cm) or TNBC breast cancer with pre-operative MRI tumor (≤ 2 cm) were found to have MRI-ALN-NPV of 90.1%, and 92.3% (92.5% in pathologic tumor ≤ 2 cm), separately, which apparently increased the NPV of 77.7% of MRI in the general population (Table 4).

The NPV is higher for MRI ≤ 3 cm than for pathology size ≤ 3 cm in nearly all molecular subtypes (Table 4). Onesti et al. and our previous study reported that MRI tumor size correlates with pathology size but tends to overestimate [40, 41]. The reason might be attributed to a smaller actual size in the MRI group than in the pathology group, which leads to less axillary involvement. Thus, using tumor size from MRI is a more favorable criterion than using size from a pathology report, which is only available post-operation. The ALN-positive rate was 29.7% in TNBC, 34.8% in ER( +)/HER-2(−), and 38.7% of HER-2( +)/[ER(−) or ER( +)] breast cancer in the current study (Table 4). Similar to our study, Lu et al. [42] and Houvenaeghel et al. [43] reported that TNBC had a lower probability of node metastasis. Owing to a high NPV and lower possibility of ALN metastasis in the TNBC group, ALN staging by MRI may be useful for the design of neoadjuvant chemotherapy or surgical planning.

Currently, the standard of ALN evaluation in invasive breast cancer patients is SLNB, and a meta-analysis showed that SLNB had a FN rate of around 8.61% (95% CI 8.05–9.2%) [38]. That means a cutoff point of NPV ≥ 90% of MRI-ALN-NPV should be the minimal requirement of the “threshold” of non-invasive ALN imaging modality to be considered the “alternative” choice compared to the current “standard of care”-SLNB. We found that by combining pre-operative clinicopathologic factors and intrinsic subtypes, MRI could identify some groups of patients with low risks of ALN metastasis, high NPVs, and low burdens of axillary disease even in FN cases (Table 4). Through the current study, we showed the possibility of improving the diagnostic accuracy (NPV) of non-invasive imaging modalities, like MRI, in a stepwise way. Recently, novel techniques such as radiomics [44] and deep-learning methods [45] showed promising results on ALN prediction, with accuracy up to 0.970. The performance of these models may be superior to radiologists, and the algorithms may become non-invasive biomarkers that contribute to the advancement of personalized medicine. However, the generalizability of deploying these AI models is still challenging.

Our present study was limited in its retrospective nature and in a single institution where all the studies were scanned on a 3 T MRI scanner. Second, we excluded patients who received neoadjuvant chemotherapy in which direct comparison of pretreatment image findings, and final nodal pathology was impossible. This may be the reason that the number of patients in TNBC and HER-2 groups was relatively small. Besides, since most suspicious nodal-positive cases would go on neoadjuvant chemotherapy nowadays, excluding NAC may have a gradually negative impact on specificity. However, it is important to note that these results are based on 2473 patients, which featured the largest number of cases reported from a single institute, with complete pre-operative breast MRI evaluation and detailed post-operative breast cancer and lymph node pathologic information. This enabled us to perform comprehensive intrinsic subtype analysis and showed stepwise improvement of ALN metastasis MRI NPV by combining clinicopathologic and imaging factors. The information and evidence derived from the current study may provide a potential for non-invasive imaging evaluation of ALN.

## Conclusion

In the current study, we demonstrated that MRI prediction of ALN metastasis differed according to different intrinsic subtypes. By combining IHC biomarkers (intrinsic subtypes) and anatomical features (tumor size), the NPV of ALN by MRI could reach more than 90% in some groups of patients. Patients with HER-2 with pre-operative MRI tumor ≤ 2 cm or TNBC breast cancer with pre-operative MRI tumor ≤ 2 cm was found to have MRI-ALN-NPV of 90.1%, and 92.3%, respectively, which apparently increased the NPV of 77.7% of MRI in the general population. By utilizing these stepwise methods, the study demonstrated that breast MRI's NPV could be enhanced, thus, making MRI a more potent non-invasive and alternative approach for pre-operative evaluation of ALN burden.

## Data Availability

The data that support the findings of this study are available on request from the corresponding author, HWL. The data are not publicly available due to their containing information that could compromise the privacy of research participants.
